# Serological evidence of rift valley fever Phlebovirus and Crimean-Congo hemorrhagic fever orthonairovirus infections among pygmies in the east region of Cameroon

**DOI:** 10.1186/s12985-018-0977-8

**Published:** 2018-04-06

**Authors:** Serge Alain Sadeuh-Mba, Gide Martial Yonga Wansi, Maurice Demanou, Antoine Gessain, Richard Njouom

**Affiliations:** 1 Virology Service, Centre Pasteur du Cameroun, 451 Rue 2005, Yaounde 2, P.O. Box 1274, Yaounde, Cameroon; 2 Unité d’Epidémiologie et Physiopathologie des Virus Oncogènes, Département de Virologie, Institut Pasteur, 28 Rue du Dr. Roux, F-75015 Paris, France

**Keywords:** Rift valley fever phlebovirus, Crimean-Congo hemorrhagic fever orthonairovirus, Seroprevalence, Pygmies, Cameroon

## Abstract

**Background:**

Rift Valley Fever Phlebovirus (RVFV) and Crimean-Congo Hemorrhagic Fever Orthonairovirus (CCHFV) specific antibodies had been documented among humans in urban settings of the southwestern and northern Cameroon in the late 1980s. Recently, evidence for enzootic circulation of RVFV was reported among livestock in both rural and urban settings in Cameroon. However, current estimates of human exposure to RVFV and CCHFV are still to be documented in Cameroon, especially in rural areas. The aim of this study was to assess the seroprevalence of RVFV and CCHFV in rural settings in the Southeastern rain forest of Cameroon.

**Results:**

Using Enzyme-linked Immunosorbent Assays, the presence of RVFV and CCHFV Immunoglobulin G antibodies was investigated in plasma samples originating from 137 Pygmies from four villages of the East region of Cameroon. The studied population was found to be 12.4% (17/137) and 4.4% (6/137) seropositive for RVFV and CCHFV, respectively. The rates of RVFV IgG were comparable between the age groups and sex. Conversely, the rate of CCHFV IgG was significantly higher among the 41–60 years old participants (*p* = 0.02).

**Conclusions:**

This study provides a substantial evidence of the circulation of RVFV and CCHFV among rural inhabitants of the East region of Cameroon.

## Background

R*ift Valley Fever* phlebovirus (RVFV), a member of the genus *Phlebovirus* in the family *Phenuiviridae*, is a mosquito-borne zoonotic virus that infects livestock and humans in Africa and the Arabian Peninsula. Clinically apparent infections are mainly observed in sheep, goats, cattle and camels in which they manifest high neonatal deaths and high rate of abortion. RVFV circulation has been repeatedly reported in domestic and wild animals in Cameroon from 1967 to 2017 based on serological [1–4] and molecular [4] evidences. With respect to humans, RVFV-specific antibodies were detected in urban populations from southwestern and northern Cameroon [5, 6].

*Crimean-Congo Hemorrhagic Fever Orthonairovirus* (CCHFV), a member of the genus *Orthonairovirus* in the family *Nairoviridae*, is characterized by tick-borne maintenance and transmission in an enzootic cycle involving ticks and mammals in endemic foci that are worldwide distributed. These foci include a wide geographic range in Western and Central Asia, the Middle East, South-Eastern Europe, and Africa. There is no apparent disease manifestation occurring in animals. However both wild and domestic animals act as reservoirs for continued tick re-infection; thus ensuring major links in the disease transmission cycle.

Reports documenting serological evidence of RVFV and CCHFV among humans in Cameroon focused only on urban settings in the southwestern and northern parts of Cameroon and were published several decades ago [5, 6]. Although RVFV have been suggested as a potential threat to human health in both rural [1] and urban [1, 5, 6] settings in Cameroon for decades, current estimates of human population exposure to RVFV are still to be determined in Cameroon, especially in rural settings. In the same manner, CCHFV specific antibodies had been “historically” reported exclusively in urban settings in Cameroon [5, 6].

## Materials and methods

**Fig. 1:**
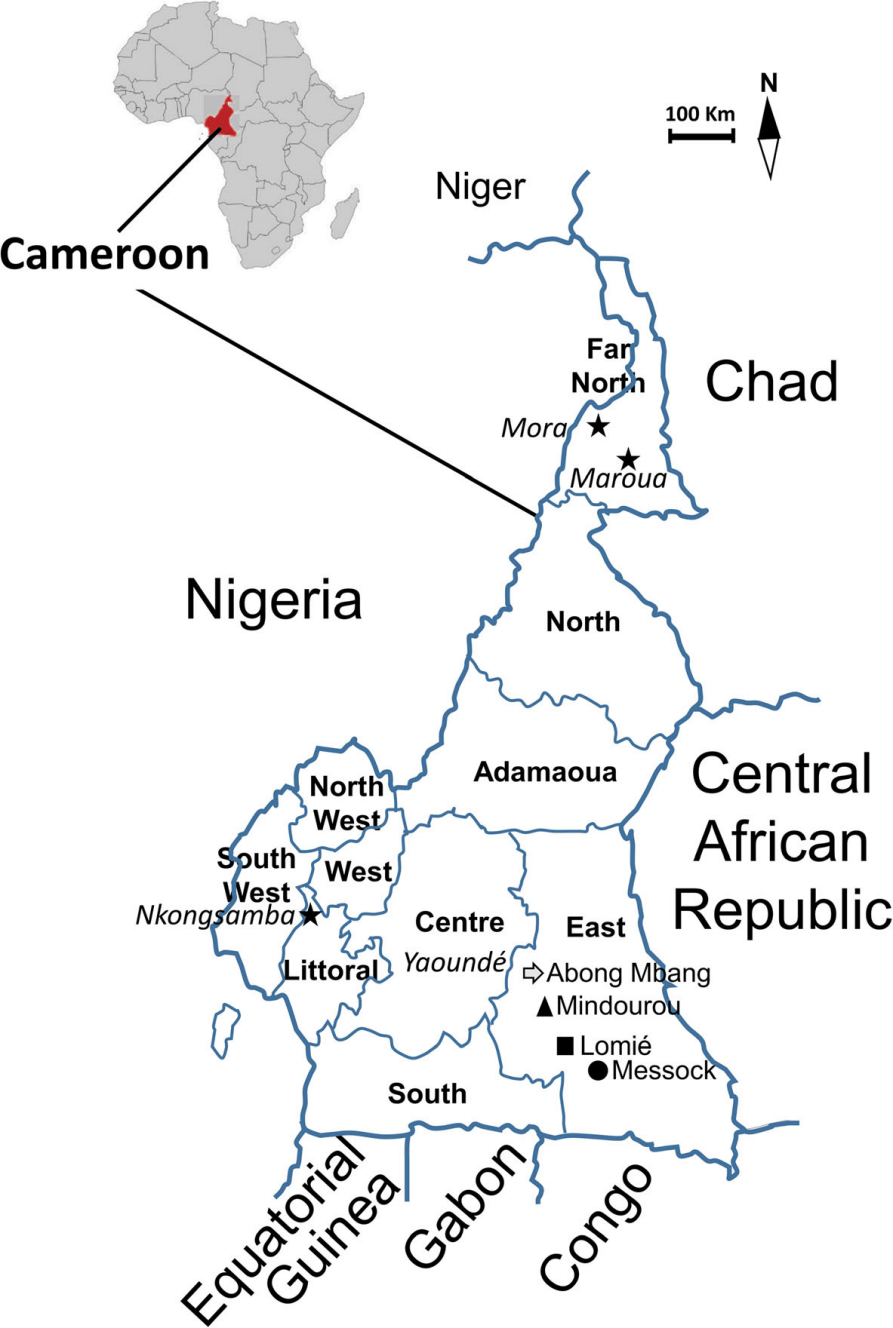
Geographic localization of the studied districts in the East region of Cameroon. Studied districts are specified by an arrow (Abong Mbang), triangle (Mindourou), square (Lomié) and circle (Messock). Urban districts (including Mora, Maroua and Nkongsamba) where previous studies were conducted among humans in Cameroon [5, 6] are indicated by stars

A cross-sectional study was carried out to assess the rate of anti-RVFV and anti-CCHFV immunoglobulin G (IgG) antibodies among the Pygmy group living in four distinct villages in the East region of Cameroon including Abong Mbang, Lomié, Messok and Mindourou (Fig. 1). The East region of Cameroon is characterized by its rain forest with dense vegetation, extensive hydrographic network, warm and humid climate and abundant precipitation. Farming and animal breeding is very limited in this region but people living in these areas may experience various degrees of exposure to Non Human Primates (NHP) and other wild animals during their lifetime, through hunting, butchering, or the keeping of animals as pets.

Plasma samples were randomly collected from 2005 to 2012 during a survey of viral emergence in Pygmies, a population that has lived in a forest environment in Cameroon for more than 20,000 years, mostly as hunter-gatherers [7–9]. The study population was made up of 137 individuals having the mean age of 44.4 ± 16.4 years and a male:female sex ratio of 1.4 (Table 1).

**Table 1:** Antibody prevalence to rift valley fever phlebovirus or Crimean-Congo hemorrhagic fever orthonairovirus among pygmies in the East region of Cameroon

Age range (years)	Sex ratio (M/F)	Average age ± SD	Number of positive samples for the specified population/no. tested (%)^a^				Total
			Abong Mbang	Lomié	Messok	Mindourou	
Rift Valley Fever Phlebovirus (RVFV)							
≤20	0.3	17.3 ± 3.7	0	0/1	0/3	0/8	0/12
21–40	2.0	32.9 ± 5.4	0/2	0/13	0/4	7/32 (21.9)	7/51 (13.7)
41–60	1.5	50.5 ± 5.5	0/4	2/12 (16.7)	0/2	5/31 (16.1)	7/49 (14.3)
61–80	1.5	69.2 ± 5.4	0/2	0/4	1/2 (50.0)	2/17 (11.8)	3/25 (12.0)
Total	1.4	44.4 ± 16.4	0/8	2/30 (6.7)	1/11 (9.1)	14/88 (15.9)	17/137 (12.4)
Crimean-Congo Hemorrhagic Fever Orthonairovirus (CCHFV)							
≤20	0.3	17.3 ± 3.7	0	0/1	0/3	0/8	0/12
21–40	2.0	32.9 ± 5.4	1/2 (50.0)	0/13	0/4	0/32 ^b^	1/51 (2.0)
41–60	1.5	50.5 ± 5.5	1/4 (25.0)	1/12 (8.3)	0/2	3/31 (9.7)	5/49 (10.2)
61–80	1.5	69.2 ± 5.4	0/2	0/4	0/2	0/17^b^	0/25
Total	1.4	44.4 ± 16.4	2/8 (25.0)	1/30 (3.3)	0/11	3/88 (3.4)	6/137 (4.4)

*RVFV* rift valley fever phlebovirus, *CCHFV* Crimean-Congo hemorrhagic fever orthonairovirus

^a^There was no significant difference between the rates of RVFV Ig G in the four studied districts (*P* > 0.05) while the rate of CCHFV Ig G was relatively higher in Abong Mbang compared to other studied districts (*P* = 0.004)

^b^CCHFV assay showed indeterminate result for a 35 years old woman and a 65 years old man originating from the district of Mindourou

Whole blood was collected in a 5-mL EDTA Vacutainer tube and centrifuged within 24 h of collection. Informed consent was obtained from adults (or from parents/legal guardians in the case of children under 21) before blood sampling. Furthermore, the participants of the study underwent a non-specific medical examination and, if necessary, were treated on site according to local procedures or were sent to local medical facilities. Enzyme-linked Immunosorbent Assays (ELISA) were used to detect RVFV and CCHFV IgG antibodies respectively in plasma samples as previously described [10, 11]. For each plate, high positive control [mean Optical Density (OD) at about 1.6] was tested in quadruplicates while the low positive control (mean OD at about 0.3) and negative control were tested in duplicates. Each serum was tested in two wells where the antigen-free cell culture supernatant (from the cell control of antigen production) was added and in other two wells where the specific antigen was added. The specific reactivity of each serum (net OD) was determined by subtracting the OD of the sample in the wells where the antigen-free supernatant was added from the OD of the same sample obtained in the wells where the specific antigen was added. The mean net OD was calculated for the quadruplicates of the high positive control serum [(OD_1_ + OD_2_ + OD_3_ + OD_4_)/4] and the reactivity of each serum specimen was calculated as percentage positivity (PP) of the high positive control serum (PP serum = 100*net OD serum/mean net OD of the high positive control). Sera with PP values > 28.98 were considered positive for RVFV-specific IgG serology assay [10, 12]. Sera with PP values > 8.5 (cut-off value equal to mean plus 2 standard deviations derived from PP values in negative sera tested in duplicates in four separate experiments) were considered positive for CCHFV-specific IgG serology assay [11].

Statistical analyzes were performed using the R software version 3.4.1. Categorical variables were described as numbers and percentages. Association between RVFV and CCHFV prevalence and other variables was assessed through Chi-2 or Fischer tests as appropriate. The threshold of significance of the *p*-values was set at 0.05.

## Results

Of the 137 plasma samples tested in this study, 17/137 (12.4%) and 6/137 (4.4%) were reactive to RVFV and CCHFV, respectively. There was no significant difference between the rates of RVFV IgG in the four studied districts (*p* > 0.05). In contrast, the rate of CCHF IgG was relatively higher in Abong Mbang compared to other studied districts (*p* = 0.004) (Table 1). Exposure to both viruses was found for only two individuals: a 47 and a 55 years old men originating from the districts of Lomié and Mindourou, respectively (Table 1). There was no significant difference between the rates of RVFV IgG obtained in the four studied age groups or between the male and female participants (*p* > 0.05). Conversely, the rate of CCHFV IgG was significantly higher among the 41–60 years old participants (*p* = 0.02). Interestingly, all six CCHFV IgG positive participants were males between 40 to 60 years old while the seventeen RVFV IgG positive participants were 28 to 75 years old.

## Discussion

This study provides substantial evidence of population exposure to RVFV and CCHFV in the eastern rain forest of Cameroon. RVFV and CCHFV exposure rates found among Pygmies in this study were higher than those originally reported at 0.25 to 1.06% among other human communities in urban settings in Cameroon and other central African countries [5, 6]. A more recent study also revealed a RVFV exposure rate of 3.3% (145/4323) among rural human populations in the neighboring Gabon [13]. A higher rate of 16.7 (56/335) was found among some people at risk, such as stock breeders, workers in slaughterhouses and livestock markets in the neighboring Central African Republic [14]. The CCHFV exposure rate of 4.4% in this study was unexpectedly higher than those originally reported as being of 0.0% to 0.5% [5, 6]. An anti-CCHFV IgG rate of 2.7% was recently reported in an urban setting in Maputo, Mozambique [15]. The relatively higher rates of the population exposure to RVFV and CCHFV, compared to those reported by “historical” studies in Cameroon [5, 6], could be associated to several factors differentiating the past and present studies. This study targeted Pygmies living in villages whereas previous studies enrolled *bantu* populations in urban areas [5, 6]. Distinct agro-ecological profiles of the study areas and/or lifestyles of the population enrolled in the present and previous studies might have also been associated to the differential rates observed. Indeed, it has been suggested elsewhere that the risk of RVFV could vary greatly with respect to the communities and the professions considered [14, 16, 17]. In contrast to RVFV whose exposure rates were comparable among groups, the rate of CCHF IgG was relatively higher among individuals from Abong Mbang compared to those from other studied districts. Since all studied participants belonged to the same community of hunter-gatherers in the rain forest of Cameroon, there is no differential behavior or lifestyle that could explain the higher CCHF exposure rate in Abong Mbang. Uniform sampling among human communities and potential vectors in both rural and urban settings is required to draw a final conclusion about the current risk and population exposure to RVFV and CCHFV infection in Cameroon.

A recent study provided molecular and serologic evidence of acute RVFV infections in livestock in Cameroon [4]. Thus it is likely that RVFV also currently affect human populations in Cameroon where testing facilities for RVFV and CCHFV are still very limited. Given that healthcare providers have limited awareness of these viruses and associated diseases, the actual impact of RVFV and CCHFV on human health is still to be documented in Cameroon. Broad species sampling among humans, livestock and wildlife as well as virological analyses of mosquitoes and ticks would be of great interest in identifying the transmission hotspot of RVFV and CCHFV in Cameroon. Such investigations would provide baseline data to set up and optimize a national RVFV and CCHFV surveillance and control in Cameroon.

## Conclusion

The seroepidemiological assessment carried out in this study provides a substantial evidence of the circulation of RVFV and CCHFV among Pygmies in some villages in the East region of Cameroon. There was no sex or age dependent association of anti-RVFV antibodies in the studied population. Anti-CCHFV IgG antibodies were observed to be detected almost exclusively among men of 40 to 60 years of age.

## Abbreviations

CCHFV: Crimean-Congo hemorrhagic fever orthonairovirus
ELISA: Enzyme-linked immunosorbent assay
IgG: Immunoglobulin G
RVFV: Rift valley fever phlebovirus
WHO: World Health Organization
